# Intergroup Contact Is Associated with Less Negative Attitude toward Women Managers: The Bolstering Effect of Social Dominance Orientation

**DOI:** 10.3390/bs13120973

**Published:** 2023-11-26

**Authors:** Federico Contu, Alessio Tesi, Antonio Aiello

**Affiliations:** 1 Department of Social and Developmental Psychology, “La Sapienza” University of Rome, 00185 Rome, Italy; 2 UniSR-Social.Lab, Vita-Salute San Raffaele University, 20123 Milan, Italy; 3 Department of Political Science, University of Pisa, 56126 Pisa, Italy; alessio.tesi@unipi.it (A.T.); antonio.aiello@unipi.it (A.A.)

**Keywords:** social dominance orientation, intergroup contact, attitudes towards women as mangers

## Abstract

This cross-sectional study examined the intergroup contact hypothesis in the workplace by enrolling 150 Italian employees. Within the framework of social dominance theory, the purpose of this study was to test the assumption that individuals with higher levels of social dominance orientation are more likely to exhibit prejudice against women in managerial positions and benefit more from intergroup contact with a female supervisor. In particular, we found that individuals with higher levels of social dominance orientation exhibited more negative attitudes towards women in manager positions, but this effect only appeared when their superiors were women, as opposed to men. In addition, participants with higher social dominance orientation experienced more positive outcomes from intergroup contact, resulting in less negative attitudes toward women managers, than those with lower social dominance orientation. Overall, these findings yield insights into how intergroup contact affects individuals with prejudice tendencies, indicating that contact with the targeted group (i.e., women in managerial positions) is negatively associated with negative attitudes towards the group, even when the prejudice is driven by social dominance orientation. These results could shed light on new routes to design practical intervention aimed at solving prejudice towards women in leadership roles.

## 1. Introduction

Identifying the processes that individuals follow in making decisions about who they want their leader to be is an important issue today [1,2]. Historically, research within social and cultural psychology has attempted to address this issue through a stereotypical, prejudice-based approach. The findings of these studies suggest that people tend to select leaders based on traditional stereotypes rather than skills or abilities [3,4,5,6]. One significant result of these biased choices is the preference for men over women in leadership roles [7]. Indeed, leadership is commonly associated with masculinity in both western and non-western societies [8,9].

Despite being studied for more than five decades, negative attitudes (i.e., prejudice) towards women leaders remain a serious hindrance in western cultures [10,11]. Therefore, the present research aimed to enrich the literature with psychological variables (i.e., individual differences) that support prejudice towards women leaders and how these factors interact with strategies that can mitigate this phenomenon. More specifically, this research examined how social dominance orientation (SDO; [12]) may interact with intergroup contact [13] in defusing negative attitudes toward women leaders.

### 1.1. The Effect of Intergroup Contact on Individual Differences

As noted above, a thriving tradition of research has linked the formation and maintenance of prejudice to variables describing individual differences [14,15,16]. Importantly, in addition to the factors that contribute to the endorsement of the stereotypical idea that women are not good leaders, previous research has also examined what factors may mitigate this type of prejudice. Specifically, the intergroup contact hypothesis posits that contact with the target of prejudice can reduce prejudice toward that particular target group [13]. Several studies have found that different types of intergroup contact, such as imagined [17,18] or extended [19], can have a positive effect on reducing intergroup prejudice. Importantly, for the present research, negative attitudes toward women leaders have also been shown to benefit from intergroup contact [20,21,22].

In particular, variables related to individual dispositional differences have been shown to enhance the effect of intergroup contact [23,24]. For example, the need for cognitive closure (NFC; [25]) magnified the positive effect of intergroup contact on prejudice (RWA; [26,27]), such as right-wing authoritarianism [28]. In other words, people with a high dispositional need for cognitive closure or right-wing authoritarianism were simultaneously found to be the most prone to prejudice and the most sensitive to intergroup contact. These findings were replicated with respect to the need for cognitive closure within prejudice against women managers. Specifically, individuals with a high need for cognitive closure showed less prejudice toward women in leadership positions (i.e., managers) when they had a woman (vs. a man) as their manager [29] and when the frequency and quality of contact with their women managers increased [30]. The present research aims to further explore the role of individual differences in moderating the effect of intergroup contact on prejudice against women in leadership roles in work organizations. Specifically, we focused on one particular individual difference, social dominance orientation (SDO) [12,31], which identifies individuals who are prone to prejudice, including stereotypes and negative attitudes toward women.

### 1.2. Social Dominance Orientation and Intergroup Contact toward Women Manager

Social dominance theory (SDT; [31]) is a widely recognized theoretical framework that seeks to comprehend the distribution of group hierarchies and inequalities in various social systems, ranging from societies [31] to specific communities and organizations [32,33,34,35]. According to the SDT, societies are fundamentally organized into hierarchical systems that favor specific groups over others. Individuals who belong to dominant groups that have greater economic, political, and social power tend to prioritize access to resources for high-status groups while impeding the distribution of resources that benefit low-status groups [31,33,36].

SDO is an individual orientation that reflects an individual’s support for group-based hierarchies, and it is an integral component of the social dominance theory [12,37]. High levels of SDO predispose individuals to support and legitimize hierarchical structures, thus reinforcing intergroup disparities [34]. As a psychological construct, SDO explains how individuals internalize and propagate societal norms that disadvantage certain groups. While members of dominant groups tend to exhibit higher SDO, even members of subordinate groups with higher SDO may internalize societal support for inequality, perpetuating the spread of group-based hierarchies (i.e., self-debilitation behaviors) [31,35]. For example, employees in various organizations, including work environments and educational settings, have been observed to comply with harsh, directive power tactics that legitimize their supervisors’ dominant positions [35]. Thus, the SDO can be understood as an individual’s orientation that captures the extent to which people of various groups support hierarchy-enhancing legitimizing myths, which are intellectual and moral justifications of inequality.

Regarding gender stereotypes, the SDT provides evidence for the existence of a “gender system” [34], which highlights that men hold a disproportionate amount of economic, political, and social resources compared to women. Individuals with higher SDO tend to view women as more suited for subordinate roles in a variety of contexts and domains [12,31]. As a result, SDO has been linked to various stereotypes and prejudices against women, including hostile sexism [38,39]. Regarding work environments, Christopher and Wojda’s [16] research has confirmed that individuals with higher SDO uphold doubtful beliefs about the idea that women, compared to men, are suited for leadership positions in men-dominated work settings. Overall, individuals with higher SDO tend to believe that women are subordinate to men in typical business situations, such as those that enhance hierarchy. Additionally, they tend to view women as more suitable for jobs that align with traditional gender roles, such as being a homemaker or engaging in caring activities [16].

As already mentioned, intergroup contact is a potent socio-psychological tool in reducing bias towards women [40]. Several studies have framed SDT to reveal that intergroup contact can interact with SDO to produce a reduction in prejudice towards subordinate groups (e.g., foreigners and immigrants) [41,42]. Overall, intergroup contact has been shown to be effective for both high and low SDO individuals [41,42,43]. Although further studies are needed, intergroup contact demonstrated to produce a higher benefit in reducing some particular forms of prejudice, such as old-fashioned racism, especially among individuals with higher levels of SDO [42]. It is reasonable to assume that intergroup contact may reduce the intergroup competitive threat (i.e., the world perceived as a completive jungle), particularly salient among individuals with higher SDO [31,33]. Specifically, the present study tests how intergroup contact would reduce negative attitudes toward women managers among individuals with different levels of (low vs. high) SDO.

### 1.3. The Present Research

Prior research has shown that individuals with higher SDO are more likely to endorse gender stereotypes and hostile sexism [16,38,39]. Thus, we expected to find the same pattern of associations in the workplace and among women in leadership roles.

**H1:**  *We hypothesized that SDO would be positively associated with negative attitudes toward women managers.*

Supporting the intergroup contact hypothesis [13], several studies have shown that intergroup contact reduces prejudice towards out-groups [40]. Additionally, intergroup contact has been found to mitigate negative attitudes towards women in leadership roles, as demonstrated by studies such as Bhatnagar and Swamy [20], Duehr and Bono [21], and Stoker et al. [22].

**H2:**  *We hypothesized that intergroup interaction with women managers would be negatively associated with negative attitudes toward women in leadership positions.*

Individual characteristics can enhance an individual’s sensitivity to the positive effects of intergroup contact [23,24]. For instance, research has found that the need for cognitive closure [26,27], right-wing authoritarianism [28], and SDO [42] can increase contact’s efficacy in reducing prejudice towards subordinate or low-status groups. We sought to expand on previous studies [29,30] that endeavored to identify individual differences that may facilitate or impede the impact of intergroup contact on reducing prejudice towards women as managers. Our study specifically examined the role of SDO as an intervening variable in moderating the effect of intergroup contact on attitudes towards women in managerial positions. Furthermore, individuals with higher levels of SDO, who are more likely to exhibit negative attitudes against women, may encounter a scenario during intergroup contact that draws attention to the disconfirmation of stereotypes against women in positions of leadership [33]. Our prediction is that especially individuals with higher (vs. lower) SDO could benefit from direct contact with women in leadership positions by reducing their negative attitudes toward these women.

**H3a:**  *We hypothesized that the positive association between SDO and negative attitudes toward women managers would be reduced when subjects had a woman (but not a male) as their supervisor.*

**H3b:**  *We hypothesized that intergroup contact with a woman (vs. man) superior would be negatively associated with prejudice towards women managers especially when the SDO levels of individuals were high (vs. low).*

## 2. Method

### 2.1. Sample Size Determination

We computed the minimum sample size required to test our hypotheses through the software G*power v3.1 [44]. Since it was the first time of testing the present hypothesis, following a conservative approach, we decided to estimate the required sample size assuming a medium effect size (*f*^2^ = 0.15). Hence, with a total of seven predictors, the power set at 0.80, and alpha = 0.05, G*power suggested a minimum N of 103 participants.

### 2.2. Participants, Design, and Procedure

Study’s hypotheses were tested trough a cross-sectional study conducted in Italy within a sample of employees (N = 150; 45.3% men, 54.7% women; *M*_age_ = 32.71, *SD*_age_ = 10.29; participants were allowed to answer “other” or “I prefer not to declare” with regard to gender, but no one answered these options). Given the nature of our hypothesis, all participants were employees (i.e., they had a superior), and 58.7% of them worked in the private sector and 41.3% worked in the public sector. The participants’ superiors were women for 35.3% and men for 64.7% of participants. With respect the educational level, the 2.7% had a middle school education or lower, 46.7% had a high school education, 48.7% had a university degree, and 2.0% of participants had a Ph.D. Participants took part in the study, on a voluntary basis, through an online procedure. More specifically, participants were contacted through social network groups (e.g., Facebook, Whatsapp, and Telegram), and they were asked to take part in research inquiring about their common attitudes towards managers within the professional field. Following ethical guidelines, participants read a text clearly explaining the study information (i.e., general purpose of the study, voluntary participation, terms of data collection, and anonymous participation), provided their informed consent, and completed an online questionnaire designed to assess basic demographic information and the research measures of interest (as described below). Participants were carefully debriefed and thanked for their participation. In addition, at the end of the questionnaire, participants had the possibility to directly contact those responsible for the research to ask for any clarification they desired. Please note that the whole questionnaire was administered in Italian. In the next section, we provide examples of items that we used translated from Italian.

The study was conducted according to the guidelines of the Declaration of Helsinki and received the Institutional Review Board approval of the department of social and developmental psychology at La Sapienza University of Rome (Prot N. 0000570).

### 2.3. Measures

**Social Dominance Orientation**: SDO was measured using the Italian adaptation [45] of the English-language SDO Scale (version 7; [37]). The Italian scale is a self-report measure composed of 16 items to which responses are given using a 7-point Likert scale (0 = strongly disagree to 6 = strongly agree). A sample item is “Some groups of people must be kept in their place.” The reliability was good (Cronbach’s *α* = 0.87).

**Attitudes Toward Women as Leaders***:* Attitudes toward women in the role of a manager were measured with the Women As Managers Scale (WAMS) developed by Peters [46]. This scale consists of 21 items, reflecting different stereotypes of women holding managerial positions (e.g., “Women are not ambitious enough to be successful in the business world”). Respondents were asked to indicate the extent to which they agreed with each statement on a 7-point Likert scale, ranging from 1 (Strong disagreement) to 7 (Strong agreement). The uni-dimensionality of the scale was extensively demonstrated [20]. Hence, the ratings of items were averaged to create an overall score for attitudes towards women managers (α = 0.89). Higher scores indicated negative attitudes towards women managers.

**Control variables:** Age, gender (−1 = men; 1 = women), education, and political orientation were included as control variables. Participants indicated their political orientation on a 7-point Likert scale, where “1” indicated an extremely left-wing orientation and “7” indicated an extremely right-wing orientation.

## 3. Results

Descriptive statistics and bivariate correlations are presented in Table 1. Among others, we highlight that SDO was positively and significantly associated with attitudes towards women as managers (*r*(149) = 0.395; *p* < 0.001). Specifically, participants who had a high SDO tended also to report high levels of negative attitudes towards women in managerial positions. Notably, the association between SDO and negative attitudes towards women managers was positive and significant for both men (*r*(67) = 0.482, *p* < 0.001) and women (*r*(81) = 0.247, *p* = 0.025). In contrast, and consistent with our hypothesis, superiors’ gender (coded as 0 = men; 1 = women) was negatively and significantly associated with attitudes towards women as managers (*r*(149) = −0.180; *p* = 0.027). Specifically, having a woman (vs. a male) as a superior was associated with less negative attitudes towards women as managers.

### 3.1. Analytical Strategy

To test our hypothesis, we tested a multiple regression model in which SDO, superiors’ gender, and the interaction between them were the main predictors. Further, participants’ age, gender, educational level, and political orientation were covariates. The analysis was performed using SPSS PROCESS macro (Model 1; [47]). Ninety-five percent CIs were employed, and 5000 bootstrapping resamples were run.

### 3.2. SDO and Attitudes toward Women as Managers: The Moderating Role of Superiors’ Gender

**Figure 1:**
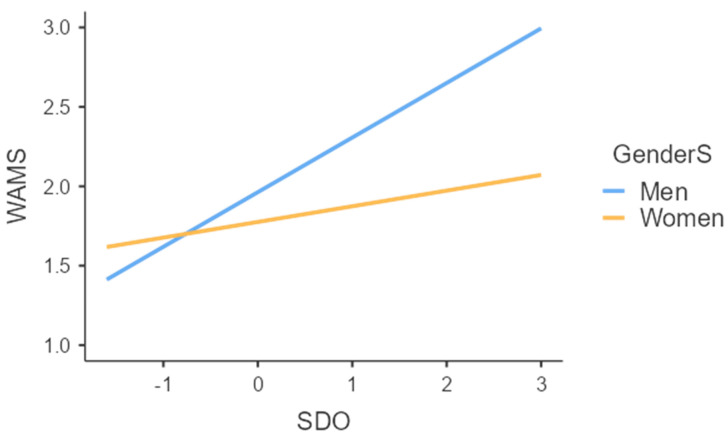
The effect of the interaction between SDO and superiors’ gender (men vs. women) on negative attitudes towards women managers. *Note: *SDO = Social Dominance Orientation; GenderS = superiors’ gender (men vs. women); and WAMS = prejudice towards women managers (a higher score indicates negative attitudes towards women managers).

**Figure 2:**
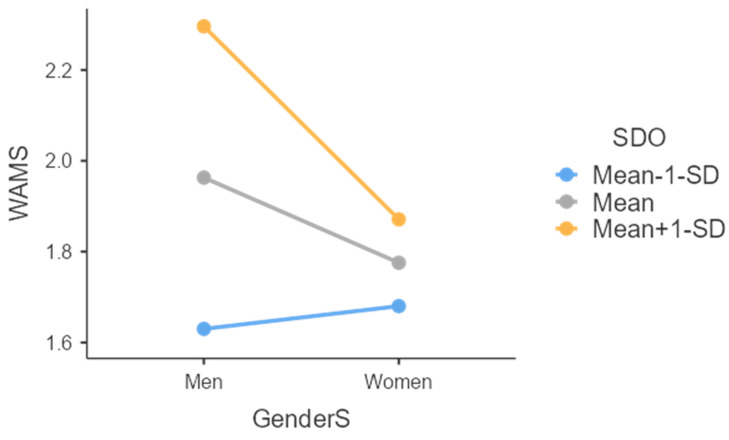
The effect of the interaction between SDO and superiors’ gender (man vs. woman) on negative attitudes towards women managers. *Note: *SDO = Social Dominance Orientation; GenderS = superiors’ gender (men vs. women); and WAMS = prejudice towards women managers (a higher score indicates negative attitudes towards women managers).

Results revealed (a) a significant and positive association of SDO (b = 0.25, SE = 0.05, *t* = 4.84, *p* < 0.001, (95%CI = 0.15; 0.35)) and (b) a barely significant association of superiors’ gender (b = −0.19, SE = 0.11, *t* = −1.74, *p* = 0.083, (95%CI = −0.40; 0.02)) with attitudes towards women managers. Thus, confirming our hypotheses (H1; H2), i.e., negative attitudes towards women managers were positively associated with SDO and negatively with having a woman (vs. a man) as a superior. Most importantly, and in line with our hypothesis (H3), the interaction between SDO and superiors’ gender were negative and significant (b = −0.24, SE = 0.10, *t* = −2.37, *p* = 0.019, (95%CI = −0.45; −0.04)). More specifically, as can be seen in Figure 1, SDO was positively associated with attitudes towards women managers only when the superiors were men (b = 0.33, SE = 0.06, *t* = 5.33, *p* < 0.001, (95%CI = 0.21; 0.46)). In contrast, as we had hypothesized, SDO had a non-significant association with negative attitudes towards women managers when the superiors were women (b = 0.09, SE = 0.08, *t* = 1.08, *p* = 0.281, (95%CI = −0.07; 0.26)). Further, when fully decomposing the interaction (Figure 2), results showed that, as hypothesized (H3), intergroup contact had a positive (i.e., reducing) effect on negative attitudes towards women managers only when SDO was high (+1SD) (b = −0.42, SE = 0.15, *t* = −2.85, *p* = 0.005, (95%CI = −0.72; −0.13)) but not when it was low (−1SD) (b = 0.05, SE = 0.14, *t* = 0.33, *p* = 0.739, (95%CI = −0.24; 0.33)). Notably, none of the covariates had a significant effect on our examined outcome (i.e., attitudes towards women managers) and, as such, the above-mentioned results remained significant even after controlling for participants’ gender, age, educational level, and political orientation.

## 4. Discussion

The persistence of gender inequality and discrimination against women is a pervasive global concern, transcending geographical, cultural, and socio-economic boundaries. In order to unravel the multifaceted layers of gender discrimination, the aim of the present study was to build on previous research [29,30] and, within the framework of the contact hypothesis [13], to further explore the role of one of the most important individual differences predicting prejudice toward various discriminated groups, namely, SDO [12,31,37]. Specifically, we hypothesized that SDO could moderate the effect of intergroup contact [40] in reducing prejudice against female managers. At its core, SDT [31,34] posits that individuals are motivated by a desire to maintain and enhance their group’s social status, which leads to the endorsement of ideologies that justify and perpetuate existing hierarchies. The theoretical framework of SDT provides a lens through which we can examine the psychological underpinnings of systemic inequalities and uncover the mechanisms that foster prejudice and discrimination against women as managers. Consistent with our hypothesis, results revealed that participants’ SDO was associated with higher negative attitudes toward women managers.

This result is consistent with previous findings [16] that people with higher SDO endorse gender stereotypes and bolster a system of inequality in which men and women are asymmetrically represented on a hierarchical ladder in terms of competence in managing a business situation [31]. Furthermore, and in line with previous findings [29,30], we confirmed that intergroup contact towards women managers was negatively associated with negative attitudes towards this target group. We strengthened these findings by highlighting that (i) participants’ SDO was positively associated with negative attitudes toward women managers only when they reported being supervised by a man (vs. woman) leader, and (ii) intergroup contact with a woman (vs. man) leader was associated with lower levels of prejudice toward women managers when participants reported higher (vs. lower) levels of SDO. Taken together, these results confirmed our expectations that people with higher SDO could benefit from intergroup contact with women supervisors and, notably, our hypotheses were validated when controlling for the effect of participants’ age, gender, educational level, and political orientation.

### 4.1. Theoretical Implications

Within the framework of SDT, the present study sheds light on the possible differential effect of intergroup contact based on people’s SDO levels. Mixed results have been found on this issue, suggesting that intergroup contact is sometimes more beneficial for those with higher SDO and sometimes for those with lower SDO, and that this difference may depend on the type of prejudice target [41,42,43,48]. Our results confirm that intergroup contact can reduce prejudice against women managers, especially for those participants with higher SDO. However, it is worth noting that the sample mean level of SDO in our research was relatively low (M_SDO_; SD = 0.97 on a 1 to 7 scale), advancing the notion that even those labeled as having a higher SDO did not overtly support social dominance, but rather they were likely more tolerant to group-based hierarchies and less inclined to support group equality. This finding contributes significantly to the SDT literature aimed at disentangling the role of SDO in facilitating or hindering the benefits of intergroup contact, highlighting that contact-based interventions are beneficial overall, even for people with low levels of SDO and, among these, those with higher levels may be more sensitive to intergroup contact. This argument is in line with a study [42] conducted with a large sample of white Americans, in which authors found that intergroup contact was similarly effective in reducing racism among participants prone to prejudice and those not prone to prejudice; the study also emphasized that intergroup contact was more effective in reducing old-fashioned anti-black racism among people with higher SDO scores and ethnic identification. People with higher levels of SDO, who are more likely to be prejudiced against women [38,39], may find intergroup contact beneficial as it could disconfirm negative attitudes, reduce competitiveness in favor of interdependence, and make the issue of discrimination against women in leadership roles particularly salient [29,33]. Furthermore, being more prejudice-prone due to higher levels of SDO exposes one to a higher perception of threat and anxiety when interacting with the target group of prejudice. Hence, prolonged contact with women in leadership roles may activate a positive spiral of de-escalation of such arousal through the disconfirmation of stereotypes and negative emotions [40,42], thereby defusing both the cognitive and the emotional component of prejudiced attitudes (cf. “affective boost”, [49]).

### 4.2. Practical Implications

What this research teaches us in terms of “practical insights” is that, on one hand, people with a high SDO are those more prone to have negative attitudes towards women managers. As such, organizations should be concerned about the fact that their employees possess such characteristics. However, on the other hand, results showed that people with higher levels of SDO also reap the maximum benefit when exposed to intergroup contact. In light of this, organizations should be aware that, when projecting interventions to reduce negative attitudes towards women managers, people that seem to be the more resistant to attitude change (because they are the most orientated to social dominance) will be also the ones on which the interventions will function better.

Notably, our results also revealed that the association between SDO and negative attitudes towards women managers was positive and significant even when controlling for the effect of participants’ gender. This result could have important practical implications. Indeed, when designing interventions in organizations, practitioners are informed to be inclusive and should include both men and women—especially those with higher SDO—in interventions that use the contact hypothesis to reduce prejudice against women in leadership positions.

### 4.3. Limitations

Regarding the limitations of the study, in line with the previous research [29], we note that we considered intergroup contact in terms of the presence or absence of a woman in a leadership position. Future research could address this limitation by also measuring the quantity and quality of contact [30]. In addition, the cross-sectional design of the study limits conclusions about causal relationships between variables. Longitudinal and experimental studies could address this limitation by tracking changes in negative attitudes toward women leaders based on intergroup contact and SDO levels. Also, future studies could implement a quasi-experimental methodology in order to rule out and control for possible intervening factors (e.g., organizational culture and climate) in determining attitudes towards (women) managers. In addition, as noted above, and consistent with other studies using analogous measures of SDO and prejudice [42,49], we found that participants on average reported relatively low scores for SDO and negative attitudes toward women (M_WAMS_ = 1.85; scale range: 1–7); thus, the results should be interpreted with some caution. Participants’ responses to self-report scales measuring prejudice and attitudes toward discrimination could be influenced by social desirability bias or reflect values typically observed in hierarchy-attenuating organizational settings where egalitarian social norms and values are consensually shared, thus hindering people’s endorsement of SDO and related attitudes [50]. To address this limitation, future studies should control for the hierarchy-enhancing or -attenuating function of the organization [32,35,51] and/or use the implicit measures of attitudes rather than self-report measures [52].

## 5. Conclusions

Despite its limitations, this study contributes to our understanding of the individual differences that may enhance the effectiveness of intergroup contact in countering gender stereotypes in leadership. By applying SDT, our results suggest that individuals with higher levels of SDO are particularly responsive to intergroup contact with women leaders. This perspective provides valuable insights for the design of contact-based interventions in organizations.

## Figures and Tables

**Table 1:** Descriptive statistics and bivariate correlations.

	SDO	GenderS	WAMS	GEND	POLITIC	AGE	M(*SD*)
SDO	−0.87						1.21(0.97)
GenderS	−0.06	-					-
WAMS	0.395 ***	−0.180 *	−0.89				1.85(0.64)
GEND	−0.151	0.365 ***	−0.177 *	-			-
POLITIC	0.248 **	−0.083	0.167 *	−0.154	-		3.57(1.53)
AGE	−0.036	0.055	0.075	−0.155	0.208 *	-	32.8(28.0)
EDU	0.044	0.202 *	−0.071	0.251 **	−0.123	−0.069	-

Note: * *p <* 0.05. ** *p* < 0.01. *** *p* < 0.001. Cronbach’s alfa is displayed in parentheses. SDO = social dominance orientation; GenderS = superiors’ gender coded as 0 = men; 1 = women; WAMS = prejudice towards women managers (a higher score indicates negative attitudes towards women managers); EDU = educational level; Politic = political orientation (higher scores indicate a right-wing orientation); and GEND = participants’ gender coded as −1 = men; 1 = women.

## Data Availability

Data are available upon request to the corresponding author.
